# Healthcare seeking behavior and antibiotic use for diarrhea among children in rural Bangladesh before seeking care at a healthcare facility

**DOI:** 10.21203/rs.3.rs-5687932/v1

**Published:** 2025-04-17

**Authors:** Sampa Dash, Mohammad Ali, Eva Sultana, Malathi Ram, Jamie Perin, Farina Naz, Bharati Roy, ABM Ali Hasan, Farzana Afroze, Fahmida Tofail, Tahmeed Ahmed, ASG Faruque, Subhra Chakraborty

**Affiliations:** International Centre for Diarrhoeal Disease Research; International Centre for Diarrhoeal Disease Research; International Centre for Diarrhoeal Disease Research; Johns Hopkins Bloomberg School of Public Health, JHU; Johns Hopkins Bloomberg School of Public Health, JHU; International Centre for Diarrhoeal Disease Research; International Centre for Diarrhoeal Disease Research; Kumudini Women’s Medical College and Hospital; International Centre for Diarrhoeal Disease Research; International Centre for Diarrhoeal Disease Research; International Centre for Diarrhoeal Disease Research; International Centre for Diarrhoeal Disease Research; Johns Hopkins Bloomberg School of Public Health, JHU

**Keywords:** Antibiotic, Bangladesh, Care-seeking, Diarrhea, Hospitalization, ORS

## Abstract

Appropriate healthcare utilization and compliance with the WHO treatment guidelines can significantly reduce diarrhea-related childhood mortality and morbidity, while overuse of antibiotics notably increases antibiotic resistance. We studied care-seeking behavior and antibiotic use for childhood diarrhea by analyzing data from 8294 diarrheal episodes of 1–59-month-old children visiting a tertiary-care hospital in rural Bangladesh. Overall, 55% of the study children received antibiotics, while only 6% had dysentery. Notably, 77% of the antibiotics were obtained from a local pharmacy without a prescription. Antibiotics alone, without zinc or ORS, were used by more children with dysentery than watery diarrhea (15% vs. 9%; p<0.001). While 85% of the children received ORS, only 7% received zinc and ORS without antibiotics. Children who received antibiotics before seeking care at the hospital had a significantly higher rate of hospitalization than those who did not have antibiotics (20% vs 13%; p<0.001). The factors that influenced the caregivers’ decision to seek care from the pharmacy were the desire for early recovery, traditional practices, faith in seeking care at pharmacies, and distance to a healthcare facility. Our findings warrant that reducing unnecessary antibiotic consumption requires increasing public awareness and strengthening laws on the sale of over-the-counter antibiotics.

## Introduction

Diarrhea is one of the principal causes of morbidity and is the third leading cause of death in children under five years old globally^1,2^. In particular, impoverished areas of the low- and middle-income countries (LMICs) in Sub-Saharan Africa, South Asia, and Latin America contribute to the majority of the global burden of under-5 diarrheal mortality. Diarrhea is also the leading cause of childhood malnutrition^1^.

Diarrhea is categorized into three clinical types, each with its specific treatments: acute watery diarrhea, bloody diarrhea, also called dysentery, and persistent diarrhea^1,2^. A variety of bacterial, viral, and parasitic organisms can cause diarrhea. According to the WHO treatment guidelines, oral rehydration solution (ORS) intake and/or intravenous (IV) electrolyte solution, depending on the severity of dehydration, along with zinc supplementation and continued feeding, are recommended for treating diarrhea in children^1–3^. Antimicrobials should not be used routinely and are recommended only for patients with bloody diarrhea (probable shigellosis), cholera, and when the patient is immunocompromised or severely malnourished^3–5^. WHO recommends against the use of antibiotics to treat acute watery diarrhea regardsless of etiology except for cholera. For children with dysentery, that is, diarrhea with blood in stool, the WHO recommended that the first-line antibiotic is Ciprofloxacin and the second-line antibiotic is Ceftriaxone^6^. Although antibiotics to treat watery diarrhea should be used only when indicated, like suspected cholera cases, they are often prescribed irrationally to patients, especially children. Injudicious and overuse of antibiotics could result in widespread resistance to antibiotics and can also lead to adverse events related to drug toxicity and detrimental effects on the gut microbiota and immune system^4,7–10^.

The global rise in antimicrobial resistance (AMR) poses a significant public health threat. Bacterial AMR was estimated to be directly responsible for 1.27 million global deaths in 2019 and contributed to 4.95 million deaths^11^. In addition, AMR has significant economic costs. The AMR could result in US$ 1 trillion in additional healthcare costs by 2050 and US$ 1 trillion to US$ 3.4 trillion gross domestic product (GDP) yearly losses by 2030^12^. A systematic review of 46 articles reported a high prevalence of antibiotic resistance to the most common first-line antibiotics in Bangladesh^13^. Studies conducted by the International Centre for Diarrheal Disease Research, Bangladesh (icddr,b) demonstrated the progression of antimicrobial resistance to *Shigella*, which is often a leading cause of morbidity and mortality in LMICs. Patients who are infected by multidrugresistant strains of *Shigella* and *Vibrio cholerae* are often subjected to more severe diseases with poor treatment outcomes^14,15^. In a recent study in Mathbaria and Chatak in Bangladesh, MDR enterotoxigenic *E. coli* strains were frequently (60% to 72%) isolated from both patients and the environment^16^.

One of the major human factors for the emergence and spread of AMR is the misuse and overuse of antimicrobials to treat, prevent, or control infections in humans, animals, and plants. A meta-analysis of demographic and health survey data from 2006–2016 of 30 countries in Sub-Saharan Africa reported antibiotic use in about 23.1% of the under-5 children for treating non-bloody diarrhea^17^. Another study conducted in Nigeria on the management practices of pediatric diarrhea revealed that 86.9% of the diarrheal cases were treated with antibiotics^18^. Only 5% of the caregivers/guardians of that study population reported that they noticed blood in the stool of their children^18^. However, that was not observed in the stool sample provided to the study healthcare facility^18^. Such overuse of antibiotics has been observed to contribute to the emergence of multidrugresistant bacteria, Methicillin-resistant *Staphylococcus aureus* (MRSA), extended-spectrum beta-lactamase (ESBL)-producing organisms, and resistant *Clostridium difficile* bacteria, leading to increased duration and severity of diarrheal episodes, longer hospital stay, increased patient mortality, and higher health-care costs^18^.

Like other LMICs, appropriate care-seeking practices for childhood diarrheal illness remain poor among Bangladeshi children. Given the expansion of unregistered healthcare centers, mostly in rural areas, unskilled health practitioners, absence of routine monitoring by the health systems, and poor access to formal healthcare services, it has become essential to understand the care-seeking practices of the rural population of Bangladesh for diarrheal disease and estimate the prevalence of the overuse of antibiotics. To control the unprecedented problem of increasing antibiotic resistance, it is also imperative to determine the factors associated with the overuse of antibiotics.

This cross-sectional study aims to understand the care-seeking patterns of under-five children with diarrhea, and determine the prevalence, sources of antibiotics, and factors associated with antibiotic use prior to hospital visits for childhood diarrheal illness among those who sought care from the pediatrics department of the Kumudini Women’s Medical College and Hospital, located in Mirzapur, Bangladesh.

## Results

### Characteristics of the study children

A total of 8294 diarrheal episodes in 1 to 59-month-old children who visited the Kumudini Hospital with diarrhea were enrolled in this study. Approximately 11% of the study children had experienced more than one diarrheal episode with a hospital visit within the study period. Among the participants, the median (IQR) age was 10 months (6–16). About two-thirds of them belonged to the age group 6 to 23 months (66.25%; 95% CI:64.25–68.29), and the majority (58%; 95% CI:56.58–58.70) of the patients were male. Among the enrolled patients, 17.05% required hospitalization (Table 1).

Regarding treatment-seeking practices before seeking care at the hospital for the diarrhea episode, 85.27% (95% CI:84.49–86.01) of the study children received ORS, and 35.17% (95% CI:34.14–36.20) received ORS and zinc together. Only 6.56% (95%, CI:6.05–7.11) of the study participants received ORS and zinc without any other medications. More than half (54.56%; 95% CI:53.60–55.75) of the study children received antibiotics at home and 78% of which had physical proof of antibiotic in the form of prescriptions and antibiotic suspension bottles. Only 5.80% of the total study population had reported passage of blood mixed with loose stool (Table 1).

### Antibiotic usage pattern

The consumption of antibiotics among children varied according to age and treatment-seeking patterns (Table 2). A total of 4535 (54.68%) children received antibiotics before seeking care from the study hospital. The median (IQR) age for those who received antibiotics was 11 months (7–16). Antibiotic use for diarrhea was found to be significantly associated with age. Among the children who received antibiotics, the majority were male (59.10%; 95% CI:57.7–60.52)]. The hospitalization rate was significantly higher among the children who received antibiotics than those who did not (20.02%; 95% CI:18.88–21.21, vs. 13.46%; 95% CI: 12.41–14.59). Zinc and ORS consumption rates were significantly higher among the children who received antibiotics.

### Comparing children with watery diarrhea and dysentery

Among the total study population, 481 children had dysentery, and the rest (7813) had watery diarrhea. Median (IQR) age of children with dysentery was 9 (6–15) months, and child with watery diarrhea were 10 (6–16) months. More children received ORS who had watery diarrhea than dysentery (85.78%; vs. 76.92%). Overall, Antibiotic use was higher among children with watery diarrhea than those with dysentery (54.91% vs. 50.94%), although the difference was not statistically significant. The use of only antibiotics without zinc and ORS, was significantly more among children with dysentery than those with watery diarrhea (15.18% vs. 9.20%). Although not significantly different, the hospitalization rate was lower among the children with dysentery compared to watery diarrhea (Table 3).

### Comparison between hospitalized and non-hospitalized children

Among the total study population, 6880 children were hospitalized (Table 4). The antibiotic consumption rate was significantly higher among hospitalized children than those who were not hospitalized (64.21%; 95% CI: 61.68–66.67 vs. 52.72%; 95% CI=51.54–53.9). On the contrary, only ORS (without any other medications) consumption rate was higher among the children who did not require hospitalization than the hospitalized diarrheal children (27.30%; 95% CI:26.26–28.36 vs. 17.54%; 95%CI=15.64–19.61). The consumption of the combination of zinc and ORS either with or without antibiotics (53.39%; 95% CI=50.79–55.98) or with antibiotics (27.86%; 95% CI=25.59–30.26) was significantly higher among the hospitalized children.

### Care-seeking pattern before seeking care at the health facility and sources of antibiotics.

**Figure 1:**
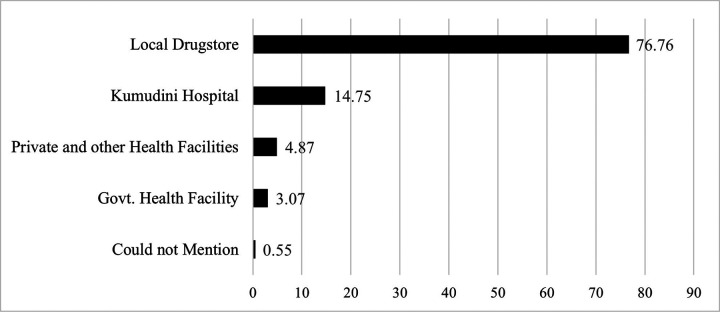
Sources of antibiotics used by the diarrheal children (n=4535) visited Kumudini Hospital

Figure 1 shows the primary sources of antibiotics consumption before coming to the hospital. The majority, comprising 76.76% of the children, sought care at a local pharmacy and received antibiotics from there. Around 3.07% of the children sought care from different levels of government health facilities, including community clinics, sub-district facilities (Upazila Health Complex), and Tangail District Hospitals, and another 4.87% of children from private and other health facilities where they were prescribed antibiotics. About 15% of the children received antibiotics from the Kumudini Hospital for the same diarrhea episode before the current visit to the hospital (Fig. 1).

### Commonly used antibiotics before seeking care at the hospital.

**Figure 2:**
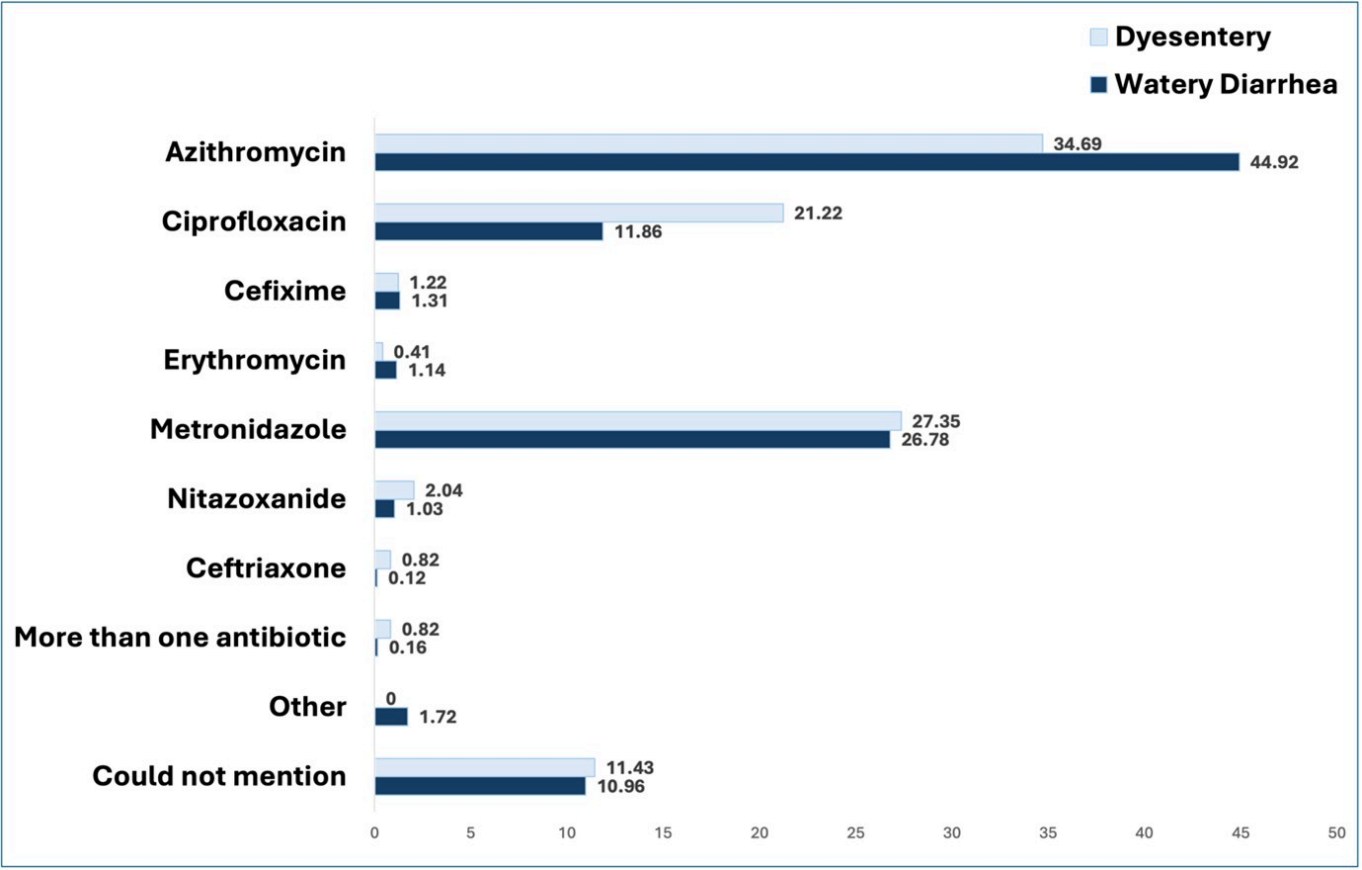
Types of antibiotics received by the under-5 children with watery diarrhea and dysentery before coming to the hospital

Among the children with dysentery who had antibiotics (n=245) prior to seeking care at the hospital, 34.69% received Azithromycin, 21.22% had Ciprofloxacin, and 27.35% had Metronidazole. Children with watery diarrhea who received antibiotics (n=4290), 44.92% of them had Azithromycin, 11.86% of them received Ciprofloxacin, and 26.78% received Metronidazole. Two children (0.16%) with dysentery and seven (0.82%) children with watery diarrhea received more than one antibiotic before seeking care at the hospital. Overall, 10.98% of the antibiotic-received children could not mention the name of the antibiotics they received. (Fig. 2).

### Antibiotic utilization rates in diarrheal disease: does access to the health care facility matters?

**Figure 3:**
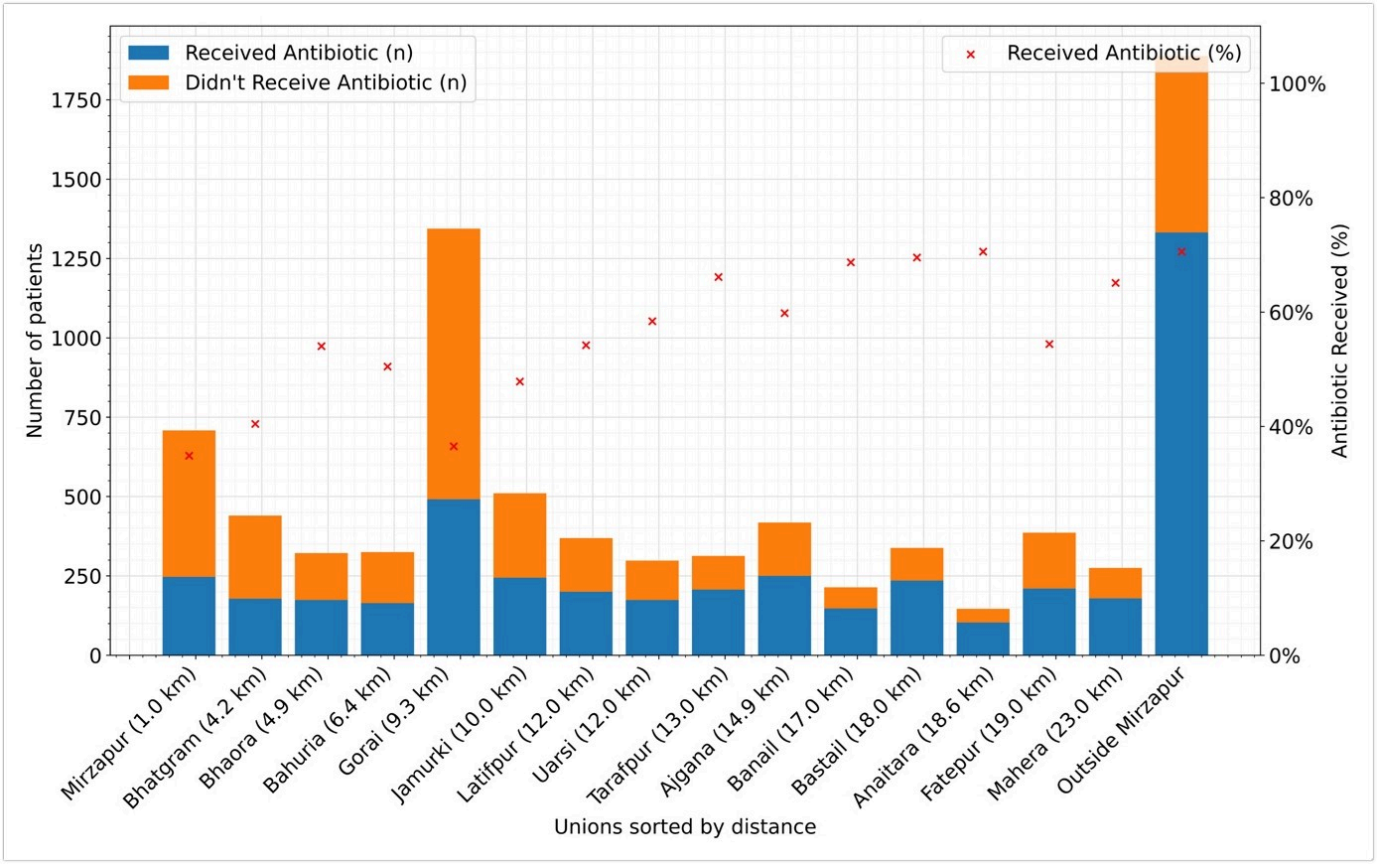
Antibiotic utilization rate among the under-5 diarrheal children visited Kumudini Hospital from March 2022 to December 2023

Children who visited the Kumudini Hospital from outside the Mirzapur sub-district showed the highest antibiotic consumption rate (70.55%). Similarly, one of the distant unions from the Kumudini Hospital is the Anaitara union (18.6km); children from that union also consumed similar rates (70.55%) of antibiotics. Among the other unions which are far from the Kumudini Hospital or any other health facility, Bastail (18.0km), Banail (17.0km), Tarafpur (13.0km), Mahera (23.0km) and Ajgana (14.9km) showed high antibiotic consumption rates (69.53%, 68.69%, 66.13%, 65.09% and 59.81% respectively). Lower antibiotic usage was found in Mirzapur (1km, 34.89%) and Bhatgram (4.2km, 40.45%), the two unions nearest to the Kumudini hospital. With the highest visiting diarrheal children, Gorai (36.53%) had a lower antibiotic consumption rate, which is only 9.3 km from the Kumudini hospital. The data on distance was collected from the data provided by Google Maps. Union is the smallest administrative area of rural Bangladesh, with an average population of 25000 (Fig. 3, Supplementary Fig. S1a, Fig. S1b online).

### Seasonal variations in antibiotic usage for diarrheal illness

**Figure 4:**
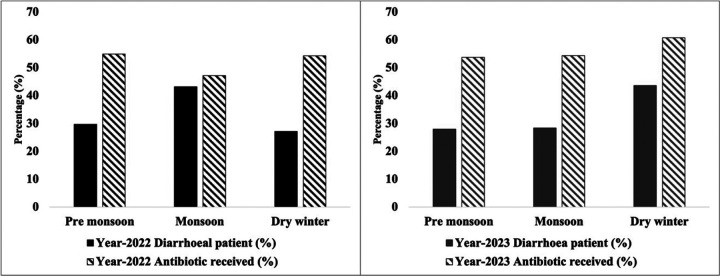
Seasonal distribution of under-5 diarrheal patients and their antibiotic consumption rate.

A distinct seasonal peak of diarrhea was observed during the study period. In the 1st year of the study period (28th March 2022 to 31st December 2022, n=3394), about 43.19% of the children with diarrhea visited during the monsoon (June-October), and 29.67% of children visited during the pre-monsoon season (March-May) (Fig. 4). During the second year of the study (1st January 2023 to 31st December 2023, n=4900), 43.73% of the study children came with diarrhea during the winter months (November-February). Children who had diarrhea during the dry winter month had 1.3 times higher odds of having antibiotics than the children who had diarrhea during the monsoon, both in 2022 and 2023 (Table 5).

### Factors associated with not seeking care from a health facility

Caregivers of the first 398 diarrheal children who consumed antibiotics without consulting a registered physician before visiting the Kumudini hospital were surveyed. The assumption that the antibiotic treatment provided by the pharmacy results in quick recovery has deterred one-third (33.67%) of the caregivers from seeking professional medical care. About 27.89% of the caregivers seek care from the local drugstore since that is their usual practice of seeking health care, and they trust the treatment provided by the pharmacy. Only for 7.04% of the caregivers, the long distance and the time required to visit a health facility were the reasons for not visiting a health facility (Table 6).

## Discussion

This study conducted with 8294 hospital visits for diarrhea over 22 months in rural Mirzapur, showed an alarmingly overuse of antibiotics for diarrhea, and these antibiotics were received mostly from the local drugstores.

According to the WHO guideline, except in the case of cholera, antibiotics should not be used for the treatment of watery diarrhea; in our study, over half of the children with diarrhea (55%) received antibiotics before visiting the health facility, while only 6% of the enrolled children had dysentery. Although the difference was not statistically significant, we found the overall use of antibiotics was higher among the children with watery diarrhea compared to those with dysentery. However, when considering the use of antibiotics alone, without ORS or zinc, children with dysentery were more likely to receive antibiotics than those with watery diarrhea. Additionally, overall ORS intake is significantly lower among children with dysentery.

These findings suggest that caregivers may perceive dysentery as a serious condition but lack awareness of the importance of concurrent use of ORS and zinc. A study conducted among infants aged 2–6 months in the capital city Dhaka in Bangladesh reported that 52% of the cases with diarrhea received antibiotics before visiting the health care facility, although the proportion of the cases that were watery diarrhea was not mentioned^19^. The WHO treatment guideline for diarrhea reserved antibiotics for specific cases of acute watery diarrhea, such as suspected cholera during an outbreak, particularly for patients who presented with profuse vomiting, severe dehydration, and acute watery diarrhea^3^. According to the national treatment guidelines of Bangladesh, the recommended antibiotics for the treatment of suspected cholera in children are Azithromycin and Ciprofloxacin^20^. In our study, we found that 45% of the children who had watery diarrhea and had antibiotics at home received Azithromycin. *Shigella* spp. is the most commonly detected pathogen in case of bloody diarrhea. For dysentery, Azithromycin is the 1st line drug of choice, and the 2nd line is Pivmecillinum and Ceftriaxone since *Shigella* spp. is the most commonly detected pathogen^20^. In our study, Azithromycin, Metronidazole, and Ciprofloxacin were the most commonly used antibiotics for children with dysentery. For dysentery, 27% of children received Metronidazole, while according to the WHO, Metronidazole is ineffective in treating shigellosis^3^.

Interestingly, contrary to the general perception that antibiotics accelerate recovery from diarrhea and reduce hospitalization, in this study, a significantly higher proportion of the children who later required hospitalization had antibiotics before visiting the hospital compared to the children who didn’t have antibiotics at home. This high hospitalization rate among the diarrheal children who had antibiotics at the onset of diarrhea could be attributed to the increased antibiotic resistance, viral cause of diarrhea, and exacerbated illness severity due to delayed adherence to the WHO diarrhea treatment guidelines, which include ORS and zinc. Notably, the children who had ORS-only had a significantly lower risk of hospitalization.

ORS and zinc are globally recognized, and WHO recommended treatments for diarrhea. In this study, among the children, while 85% received ORS, only 35% received zinc and ORS in combination with or without other medications at home. According to the Bangladesh Demographic and Health Survey (BDHS), 2022, 76% children received ORT (Oral rehydration therapy), that is, ORS or recommended homemade fluids, including 74% received ORS and 43% of the children received both zinc and ORS^21^ which differed from that reported in the BDHS 2007, only 20% children received both ORT and Zinc while 81% received only ORT^22^. These findings signify a noteworthy increase in the use of ORS but lower zinc use among children with diarrhea. There is a further need for increasing public awareness along with increased implementation and affordability of community-based health programs to use ORS and zinc in the management of diarrhea.

In this study, 77% of those who received antibiotics, purchased antibiotics from the local drugstores without presenting any formal prescriptions from a healthcare professional which was higher than 44%, reported by the BDHS 2017–18. This practice indicates a significant weakness in the health system to educate, incentivize, motivate, and regulate the salesmen of local drugstores to promote rational antibiotic use^23^. Notably, 23% of the study children who received antibiotics for watery diarrhea were treated at different health facilities, highlighting a major gap between the knowledge and practices among healthcare professionals. This study also observed a higher antibiotic usage rate during winter and at this time of the year the prevalence of viral diarrhea increases which indirectly shows that children with viral diarrhea are receiving antibiotics.

Our study found that the major reasons for the caregivers to seek care from a local pharmacy are the perception of diarrhea as a mild disease, the traditional practice of seeking care from a local pharmacy, and faith that the drugs prescribed by the pharmacy will help early recovery. Long waiting times at the health facilities and the lack of family support to take the ill child to the health facility also played a role in demotivating them to seek care from a professional healthcare facility. This study demonstrates that in addition to increasing public awareness of the drawbacks of the current practices of seeking care at the local pharmacy, caregivers’ perceptions of diarrhea and its effects on their children’s health need to be changed.

We also analyzed whether the distance from the health care facility is a factor in seeking care at the local pharmacy and consuming antibiotics. Although only 7% of the caregivers indicated distance as the reason for not seeking care at the registered health facilities, our analysis of the areawise distribution of the diarrheal children reporting to the Kumudini Hospital and their antibiotic usage rates showed a different scenario. Children from distant areas were the least likely to visit healthcare facilities and also consumed the most antibiotics. For the children who came to the Kumudini Hospital from adjacent communities, the reverse pattern was observed. This signifies that the government and national and international NGO-facilitated health promotion programs are least reached by the people of remote areas, contributing to low levels of appropriate health knowledge and suboptimal care-seeking behavior among the local population.

This study has several strengths. Data were collected from a large number of study children presenting with diarrhea over almost two years. There was an unbiased selection of the study population, which represents the children of rural Bangladesh, since the data was collected from the tertiary level hospital of rural Bangladesh. The study analyzed the care-seeking behavior among the caregivers for diarrhea management as well as quantified the overuse of antibiotics. Additionally, it assessed the factors associated with antibiotic overuse. This study also has limitations. Our study was only focused on the children who visited the hospital for their diarrhea illness and thus failed to quantify antibiotic use at the community level for mild diarrhea cases that were not reported at the hospital. Furthermore, the study did not investigate the socio-economic profile of the study children, which could influence the decision of the caregivers for healthcare seeking and antibiotic use.

## Conclusion

Our study shows that there was a significant overuse of antibiotics for watery diarrhea in rural Bangladesh, which was associated with the perception of diarrhea illness and care-seeking behavior among the caregivers, along with the practices of the local pharmacies. In addition, access to healthcare facilities also influenced the decision-making of healthcare seeking and impacted antibiotic use. This study also reveals that consuming antibiotics at the onset of all-cause watery diarrhea didn’t lower the risk of hospitalization; in fact, the risk was higher, which warrants the need for cause-specific and AMR-specific antibiotic treatment. The findings from this study on unreasonable antibiotic use for diarrheal illness warrant strengthening laws and regulations on the sale of over-the-counter antibiotics as well as raising public awareness regarding the impact of diarrhea and the consequences of overuse of antibiotics on health.

## Methodology

### Ethical Consideration

The INSIGHT study protocol^24^ was reviewed and approved by the Institutional Review Board of the Johns Hopkins Bloomberg School of Public Health and the Ethical Review Committee of the icddr,b. Informed voluntary written consent from the parents/caregivers was obtained. All the data collection methods were performed in accordance with the Declaration of Helsinki.

### Study Site

Kumudini Women’s Medical College and Hospital (Kumudini Hospital) is a tertiary-level health facility in Mirzapur Upazila, Tangail. Mirzapur is one of the sub-districts of Bangladesh, situated about 54 km northwest of Dhaka, the capital city of Bangladesh. It is home to a population of approximately 410,000 and comprises an area of 374 square kilometers. Kumudini Hospital is one of the oldest and largest (750-bed) health facilities in rural Bangladesh. This hospital is pivotal in providing essential medical care to the local community and beyond. It is the only tertiary care hospital in Mirzapur. It has a well-established pediatric department that treats approximately 7500 children every month, both in outpatient and inpatient. A diarrhea treatment unit started in 1982 and serves nearly 6000 diarrhea patients yearly. Despite the Kumudini Hospital being a private health facility, it has a vast capacity to treat patients with minimal charges and at free of cost, especially for those who cannot afford the treatment.

### Operational definition

Diarrhea: Diarrhea is defined as the passage of three or more loose/liquid stools within 24 hours period.

Dysentery: Any diarrheal episode in which any loose or watery stool contains visible blood^1,2^.

### Study Design

A cross-sectional study design was followed among the 1–59-month-old children who visited the pediatric department of the Kumudini Hospital in Mirzapur and had diarrhea. This study was conducted under the parent study, Impact of Non-Dysentery *Shigella* Infection in Growth and Health of Children over Time (INSIGHT)^24^. Approximately 350 diarrheal children under five years old, on average, visit the pediatric department of this facility every month, and the majority are from around 15 miles (0–23km) from the facility. Upon arrival, the children first received the required care from the hospital’s attending physicians. The INSIGHT study staff then approached the caregiver of each child with a study eligibility flyer. The eligible caregivers who gave informed, voluntary consent were administered a pre-validated questionnaire including age, gender, address, type of diarrhea, treatment including antibiotics received before coming to this facility, and the source of the antibiotics.

### Data collection

The data for this study was collected from diarrheal events of 1–59-month-old children visited Kumudini Women’s Medical College and Hospital with diarrhea from March 2022 to December 2023. A diarrheal episode with a hospital visit was considered as one event. Approximately 11% of the study children had experienced more than one diarrheal episode with a hospital visit within the study period. An episode was considered new when there were three or more days without diarrhea following the previous episode. The study staff collected data 7 days a week from 7 am to 9 pm. Data was collected in paper forms and was later transferred to the study electronic database by the study staff every day.

We also conducted a survey to identify the factors that influenced caregivers to refrain from seeking appropriate medical care from a health facility in the health system of Bangladesh. We selected the first 398 caregivers of under-five children with diarrhea who received antibiotics without consulting a qualified physician or visiting a health facility. They were asked about their reason for seeking care from unqualified and unauthorized healthcare practitioners including salesmen of pharmacy, quack, etc. A structured questionnaire was administered, listing possible reasons for their choice. If anyone mentioned any reason that was not listed in the data collection tool, it was noted in the “Others” category and analyzed accordingly. If a caregiver provided multiple reasons, we asked them to identify the primary reason that topped the list and had the greatest influence on their decision. Only that reason was considered.

### Statistical analysis

The data were analyzed using STATA version 15.0 (Stata Corp, College Station, Texas, USA). Frequency/proportion was used to document the study population’s characteristics. Continuous data (age of the children) were presented as median and IQR as the data were not normally distributed, while categorical variables were represented as counts and percentages. Non-parametric Mann-Whitney test for the continuous data and bivariate analysis using Pearson’s Chi-square test of independence for categorical variables were used to understand the association of independent and outcome variables. We performed logistic regression to find the association between seasons and antibiotic consumption. The statistical significance of the bivariate analysis was determined when p-value < 0.05.

The outcome variables of the study were the prior use of antibiotics for diarrheal illness before reporting to the hospital, required hospitalization, and dysentery. The question on antibiotic usage at home was asked to the caregiver to reveal whether the child was given any antibiotic for this episode of diarrhea within the last three days before the hospital visit. The caregivers were asked to show physical proof (if available) like the prescription or the drug suspension bottle, or strip or cover of the medicine or describe the medicine (color, liquid, or pills, dosage, price, formulation, and whether the provider advised the mixing of any contents of the bottle with water) to confirm that the reported drug is an antibiotic. If they could not show any physical proof of received antibiotics but could recall from memory, that history was taken and documented. To determine dysentery cases, caregivers were asked if the child passed any loose stool with blood. Requirements of the hospitalization were determined by checking the prescription provided by the attending physician.

The common care-seeking practices were determined by the use of antibiotics, source of antibiotics, consumption of ORS, and zinc before visiting the hospital. The covariates were age, sex, date of visit, hospitalization required or not, source of antibiotics, consumption of ORS, and zinc before visiting the hospital, address, and demographic profile. Seasons were defined as the dry winter season, from November to February; the pre monsoon season, from March to June; and the monsoon season, from June to October.

## Figures and Tables

**Table 1.** Characteristics of the study children and medications received before seeking care at the hospital for the diarrhea episode

Total study participants (N=8294)	n (%)	95% CI
Age of the children (months)		
Median (IQR)	10 (6–16)	
1–5	1739 (20.97)	20.11–21.86
6–11	3021 (36.42)	35.40–37.47
12–23	2474 (29.83)	28.85–30.82
24–59	1060 (12.78)	12.08–13.52
Male children	4781 (57.64)	56.58–58.70
Children with dysentery	481 (5.80)	5.32–6.32
Children required hospitalization	1414 (17.05)	16.25–17.87
Medications received before seeking care at the hospital		
Any ORS	7072 (85.27)	84.49–86.01
Only ORS	2126 (25.63)	24.7–26.58
Any zinc	3067 (36.98)	36.00–38.02
Only zinc	48 (0.58)	0.44–0.77
Any zinc and ORS	2917 (35.17)	34.14–36.20
Zinc and ORS only	544 (6.56)	6.05–7.11
Any antibiotic(s)	4535 (54.68)	53.60–55.75
Only antibiotic(s)	792 (9.55)	8.94–10.2
Received antibiotics from other than a healthcare facility	3502 (42.2)	41.16–43.29

**Table 2.** Characteristics of the children and medications associated with antibiotic use before seeking care at the hospital for the diarrhea episode and requirement of hospitalization.

Total children N=8294	Without antibiotic		Antibiotic received		*p-value*
	n=3759		n=4535		
	n (%)	95%CI	n (%)	95% CI	
Age of the children (months)					
Median (IQR)	9 (5–16)		11 (7–16)		<0.001^1^
1–5	946 (25.17)	23.8–26.58	793 (17.49)	16.41–18.62	
6–11	1321 (35.14)	33.63–36.68	1700 (37.49)	36.09–38.91	
12–23	1007 (26.79)	25.4–28.23	1467 (32.35)	31.00–33.72	
24–59	485 (12.90)	11.87–14.01	575 (12.68)	12.74–13.68	

Male	2101 (55.89)	54.3–57.47	2680 (59.10)	57.7–60.52	0.003^2^

Hospitalization	506 (13.46)	12.41–14.59	908 (20.02)	18.88–21.21	<0.001^2^

Medications received before seeking care at the hospital					
Any ORS	2910 (77.41)	76.05–78.72	4162 (91.78)	90.94–92.54	<0.001^2^
Any Zinc	795 (21.15)	19.87–22.48	2272 (50.10)	48.64–51.55	<0.001^2^
Any Zinc and ORS	743 (19.77)	18.52–21.07	2174 (47.94)	46.49–49.39	<0.001^2^

1 Non parametric Mann-Whitney U test was used to determine significance.

2 Chi-square test was used to determine significance.

**Table 3.** Comparison of characteristics and medication used between the children with watery diarrhea and dysentery before seeking care at the hospital.

Total children N=8294	Watery diarrhea		Dysentery			*p-value*
	n=7,813		n=481			
	n (%)	95% CI	n (%)		95% CI	
Age of the children (months)						
Median (IQR)	10 (6–16)		9 (6–15)			0.0162^1^
1–5	1635 (20.93)	20.04–21.84	104 (21.62)		18.17–25.53	
6–11	2818 (36.07)	35.01–37.14	203 (42.20)		37.86–46.67	
12–23	2363 (30.24)	29.24–31.27	111 (23.08)		19.53–27.06	
24–59	997 (12.76)	12.04–13.52	63 (13.10)		10.37–16.42	

Male	4500 (57.60)	56.5–58.69		281 (58.42)	53.96–62.75	0.723^2^

Hospitalization	1343 (17.19)	16.37–18.04		71 (14.76)	11.86–18.22	0.169^2^

Medications received before seeking care at the hospital						
Any ORS	6702 (85.78)	84.99–86.54		370 (76.92)	72.94–80.47	<0.001^2^
ORS only	2009 (25.71)	24.76–26.69		117 (24.32)	20.7–28.36	0.498^2^
Any Zinc	2895 (37.05)	35.99–38.13		172 (35.76)	31.6–40.15	0.568^2^
Zinc only	43 (0.55)	0.41–0.74		5 (1.04)	0.44–0.77	0.17^2^
Any Zinc and ORS	2758 (35.30)	34.25–36.37		159 (33.06)	28.99–37.39	0.317^2^
Zinc and ORS only	519 (6.64)	6.11–7.22		25 (5.20)	3.54–7.58	0.214^2^
Any antibiotic	4290 (54.91)	53.8–56.01		245 (50.94)	46.47–55.38	0.089^2^
Only antibiotic	719 (9.20)	8.58–9.86		73 (15.18)	12.24–18.67	<0.001^2^
Antibiotic+Zinc+ORS	1552 (19.86)	18.99–20.76		83 (17.26)	14.13–20.9	0.163^2^

1 Non parametric Mann-Whitney U test was used to determine significance.

2 Chi-square test was used to determine significance.

**Table 4.** Comparison between the children who required hospitalization and those who did not.

Total children N=8294	Non-hospitalized diarrheal children		Hospitalized diarrheal children		*p-value*
	n=6880		n=1414		
	n (%)	95%CI	n (%)	95%CI	
Age of the children (months)					
Median (IQR)	10 (6–16)		11 (7–19)		<0.001^1^
1–5	1568 (22.79)	21.81–23.8	171 (12.09)	10.49–13.9	
6–11	2472 (35.93)	34.8–37.07	549 (38.83)	36.32–41.4	
12–23	2031 (29.52)	28.45–30.61	443 (31.33)	28.96–33.8	
24–59	809 (11.76)	11.02–12.54	251 (17.75)	15.85–19.83	

Male	3932 (57.15)	55.98–58.32	849 (60.04)	57.46–62.57	0.045^2^

Medications received before seeking care at the hospital					
Any ORS	5764 (83.91)	83.03–84.76	1304 (92.22)	90.7–93.51	<0.001^2^
ORS only	1878 (27.30)	26.26–28.36	248 (17.54)	15.64–19.61	<0.001^2^
Any Zinc	2296 (33.37)	32.27–34.5	771 (54.53)	51.92–57.11	<0.001^2^
Zinc only	44 (0.64)	0.48–0.86	4 (0.28)	0.11–0.75	0.107^2^
Any Zinc and ORS	2162 (31.42)	30.34–32.53	755 (53.39)	50.79–55.98	<0.001^2^
Zinc and ORS only	423 (6.15)	5.60–6.74	121 (8.56)	7.21–10.13	0.001^2^
Any antibiotic	3625 (52.72)	51.54–53.9	908 (64.21)	61.68–66.67	<0.001^2^
Only antibiotic	237 (3.44)	3.04–3.90	30 (2.12)	1.49–3.02	0.01^2^
Antibiotic+Zinc+ORS (only)	1241 (18.04)	17.15–18.96	394 (27.86)	25.59–30.26	<0.001^2^

1 Non parametric Mann-Whitney U test was used to determine significance.

2 Chi-square test was used to determine significance.

**Table 5.** Seasonal association of antibiotic consumption in 2022 and 2023 among the under-5 diarrheal children visited Kumudini hospital. Logistic regression analysis was performed to find the OR, CI, and p-value.

	Year-2022			Year-2023		
Season	Unadjusted OR	95% CI	*p-value*	Unadjusted OR	95% CI	*p-value*
Monsoon	Reference			Reference		
Pre monsoon	1.36	1.16–1.60	<0.001	0.97	0.84–1.13	0.725
Dry winter	1.32	1.12–1.56	0.001	1.3	1.13–1.49	<0.001

**Table 6.** Reasons for not seeking care from a healthcare facility for the diarrheal episode.

Reason for not visiting a health facility	N (398)	%
Perception of diarrhea as a mild illness by the caregiver	20	5.03
Perception that pharmacy provided treatment will result early recovery	134	33.67
Habit of seeking treatment from local pharmacy	111	27.89
Living far from a health facility	28	7.04
Lack of family support	46	11.56
Long waiting time at the health care facility	59	14.82

## Data Availability

All data generated or analysed during this study are included in this published article and its Supplementary Information files. Any additional data related to this paper are available upon request to the corresponding author, Email: schakr11@jhu.edu
